# Supplementary material for: Freezing solute atoms in nanograined aluminum alloys via high-density vacancies
Source: Nat Commun. 2022 Jun 17;13:3495. doi: 10.1038/s41467-022-31222-6 (PMC9206034; doi:10.1038/s41467-022-31222-6)
Supplement: Supplementary file 1 — Supplementary Information [file 41467_2022_31222_MOESM1_ESM.pdf]

Supplementary Information for Freezing solute atoms in nanograined aluminum alloys via high-density vacancies

Shenghua Wu[1], Hanne S. Soreide[2], Bin Chen[3], Jianjun Bian[4], Chong Yang[1], Chunan Li[2], Peng Zhang[1], Pengming Cheng[1], Jinyu Zhang[1], Yong Peng[3], Gang Liu[1]*, Yanjun Li[2]*, Hans J. Roven[2]*, Jun Sun[1]*

1. State Key Laboratory for Mechanical Behavior of Materials, School of Materials Science and Engineering, Xi'an Jiaotong University, Xi'an 710049, China
2. Department of Materials Science and Engineering, Norwegian University of Science and Technology, 7491 Trondheim, Norway
3. Key Laboratory of Magnetism and Magnetic Materials of the Ministry of Education, School of Physical Science and Technology and Electron Microscopy Centre of Lanzhou University, Lanzhou University, Lanzhou 730000, China
4. Department of Industrial Engineering, University of Padova, Via Gradenigo 6/a, Padua, 35131, Italy

* Corresponding authors: lgsammer@xjtu.edu.cn (GL), yanjun.li@ntnu.no (YJL), hans.j.roven@ntnu.no (HJR), junsun@xjtu.edu.cn (JS)

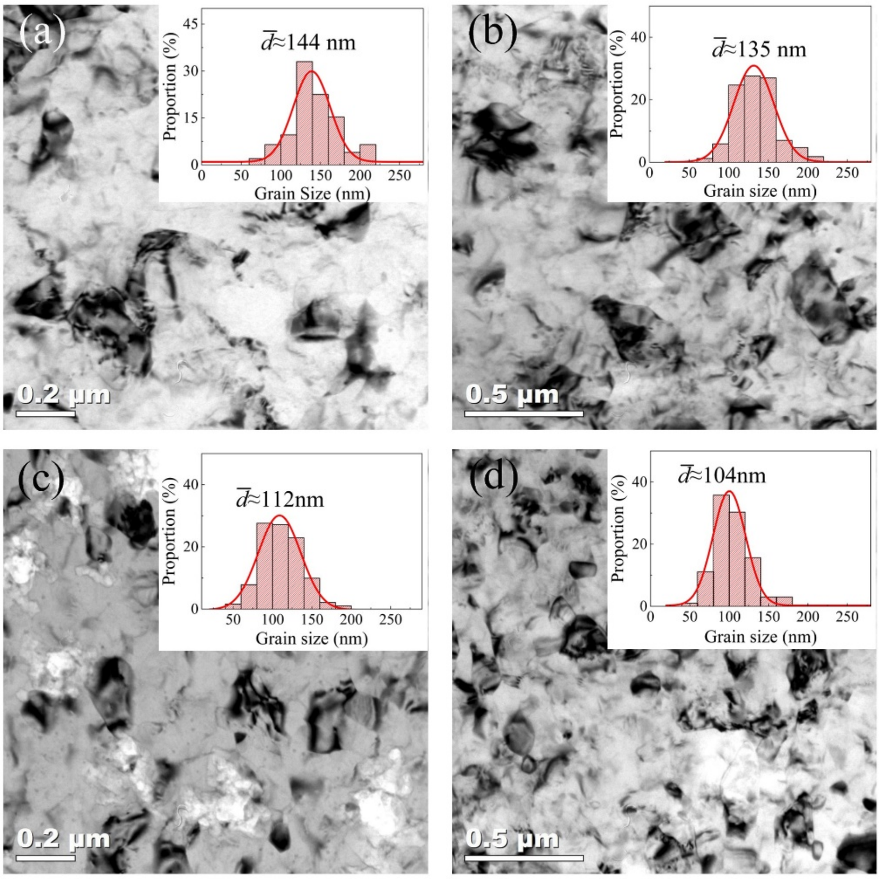

Supplementary Figure 1 | Deformation structure and grain size distribution after HPT. Typical bright-field TEM images showing the grains in **a,** AlCu-R. **b,** AlCuSc-R. **c,** AlCu-C. **d,** AlCuSc-C alloys. Insets are the corresponding statistical histograms of grain size distribution, where $\bar{d}$ is the average grain size.

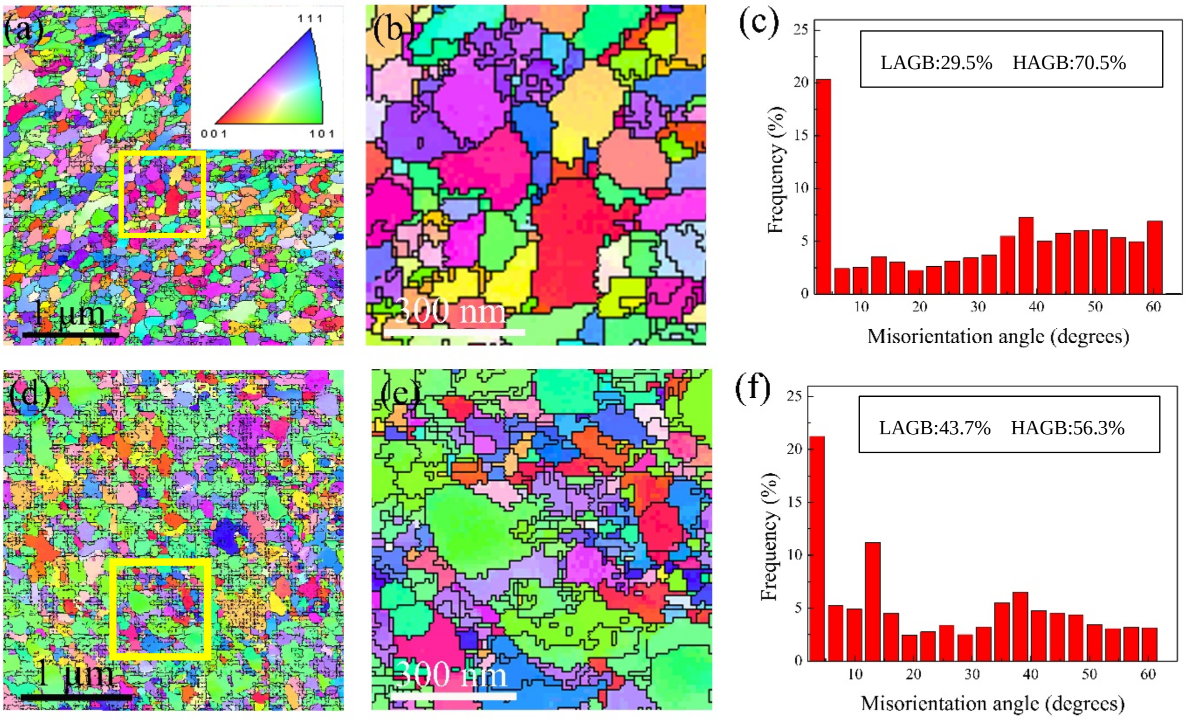

Supplementary Figure 2 | Orientation map and misorientation distribution of grain boundaries in the AlCu-R and AlCuSc-C alloys. a, Typical ASTAR-TEM orientation map of the AlCu-R alloy. **b,** Magnified image of the square area in **a**. **c,** The corresponding misorientation distribution of grain boundaries in the AlCu-R alloy, where the proportions of low angle grain boundaries (LAGBs) and high angle grain boundaries (HAGBs) are presented. **d,** Typical ASTAR-TEM orientation image of the AlCuSc-C alloy. **e,** Large magnification of the square area in **d**. **f,** The corresponding misorientation angle distribution of grain boundaries in the AlCuSc-C alloy.

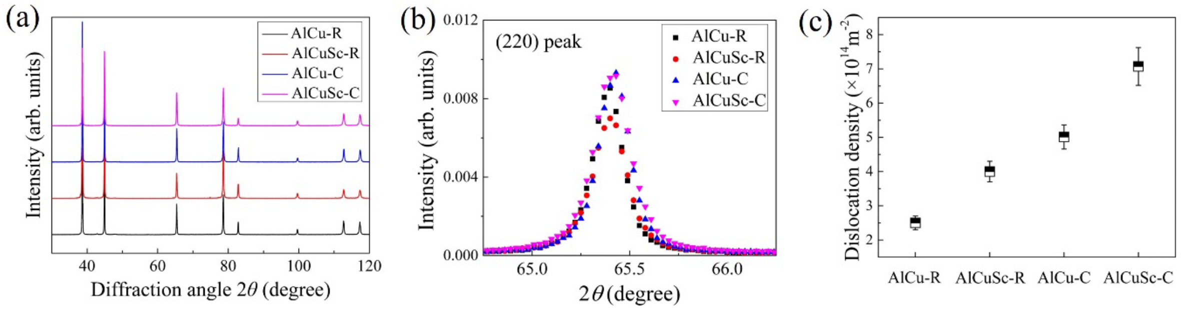

Supplementary Figure 3 | Synchrotron XRD patterns and the calculated dislocation density in the AlCu-R, AlCuSc-R, AlCu-C and AlCuSc-C alloys. a, Synchrotron XRD patterns. **b,** Representative diffraction peak of $(220)_{Al}$. **c,** Dislocation density calculated by using the Convolutional Multiple Whole Profile (CMWP) procedure. The error bars represent standard deviations from the mean for sets of three tests. The more significant broadening of $(220)_{Al}$ peak of the AlCuSc-C alloy than the other alloys indicates that it has a higher dislocation density due to the minor Sc addition and HPT at liquid nitrogen temperature.

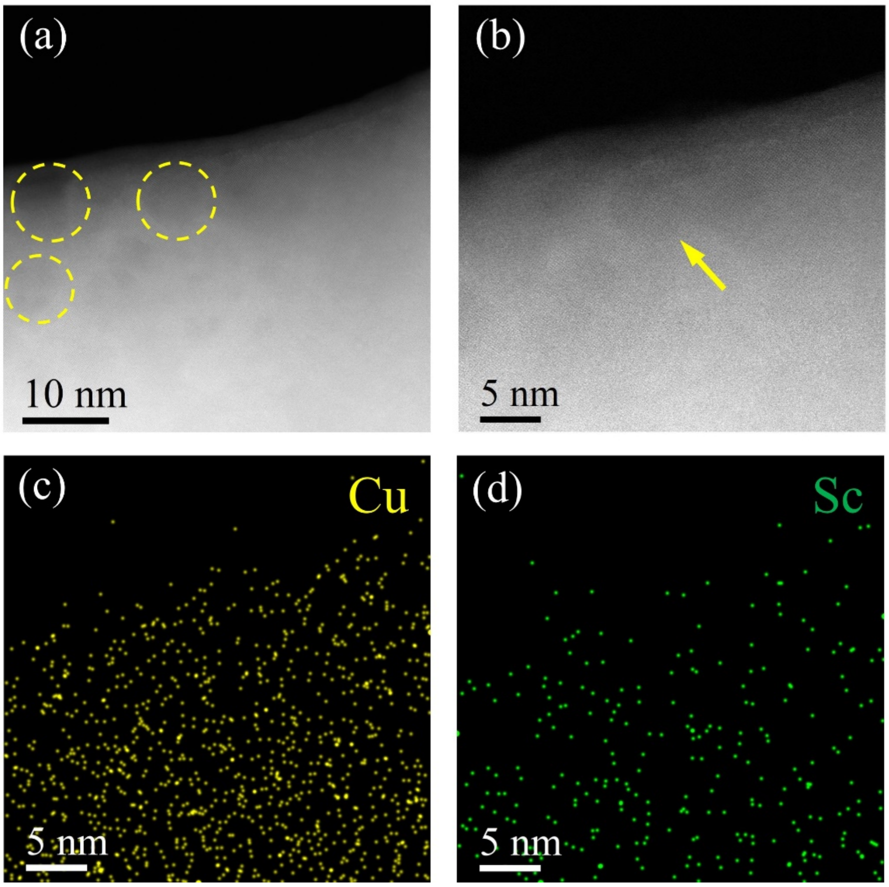

Supplementary Figure 4 | The voids in the AlCuSc-C alloy after preparation by ion milling. a, HAADF-STEM image viewed along <100>$_{Al}$ showing the voids as marked by the dashed circle. Representative HAADF-STEM image (**b**) and corresponding Cu (**c**), Sc (**d**) elemental mapping showing no Cu or Sc segregation in the voids.

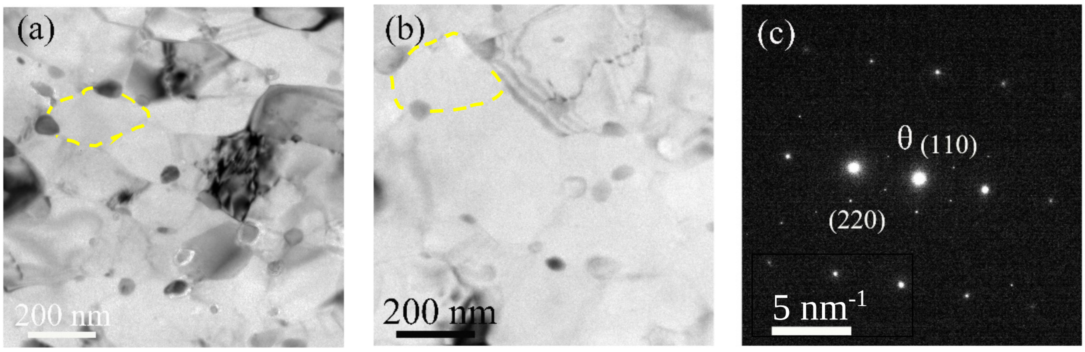

Supplementary Figure 5 | Typical bright-field TEM images of the AlCu-R (a) and AlCu-C (b) alloys aged at 125°C for 3h and selected area electron diffraction (SAED) pattern of the AlCu-R alloy (c). a and **b** show that a large quantity of θ precipitates have formed in the two alloys. **c,** SAED pattern confirms the formation of θ precipitates.

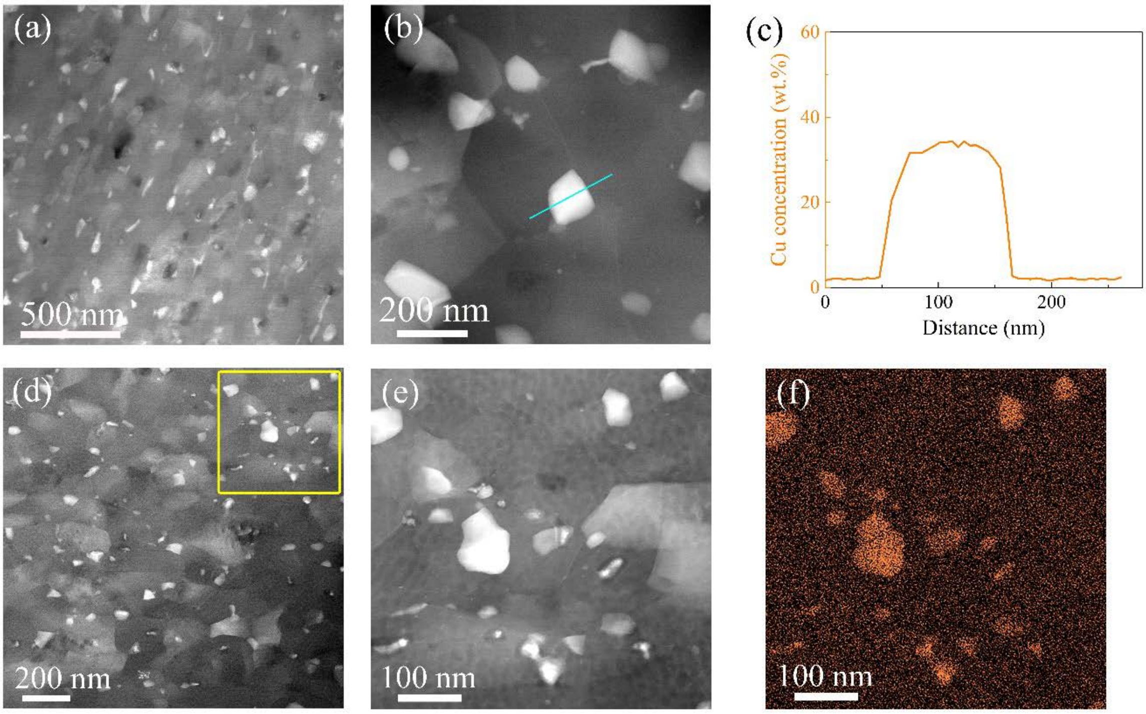

Supplementary Figure 6 | Typical HAADF-STEM images and EDS results of the AlCu-R, AlCu-C and AlCuSc-R alloys stored at room temperature for 6 months. a and **b** show a large quantity of intergranular θ precipitates in the AlCu-R and AlCu-C alloys, respectively. **c,** The EDS line scanning result of a θ precipitate corresponding to the blue line labelled in **b**. **d** shows a large quantity of intergranular θ precipitates and some intragranular θ precipitates in the AlCuSc-R alloy. **e** and **f**, Typical HAADF-STEM image and corresponding elemental mapping of Cu obtained from the framework in **d**.

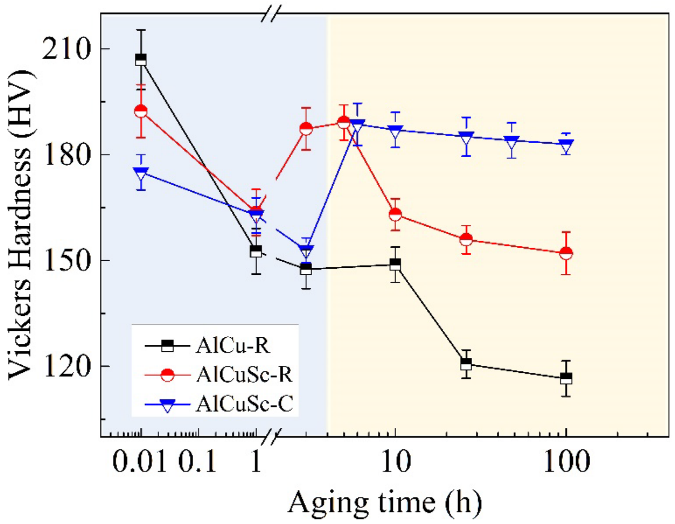

Supplementary Figure 7 | The hardness evolution with aging time at 125°C for the AlCu-R, AlCuSc-R and AlCuSc-C alloys. The error bars represent standard deviations from the mean for sets of three tests.

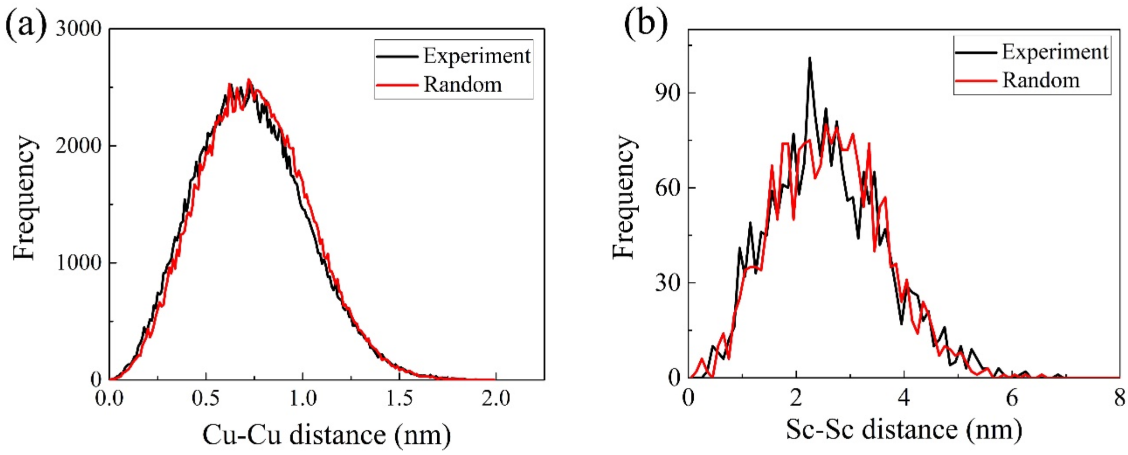

Supplementary Figure 8 | The nearest neighbor distance distribution profiles of Cu and Sc atoms in the AlCuSc-C alloy after natural aging for 6 months. a, Cu. **b,** Sc.

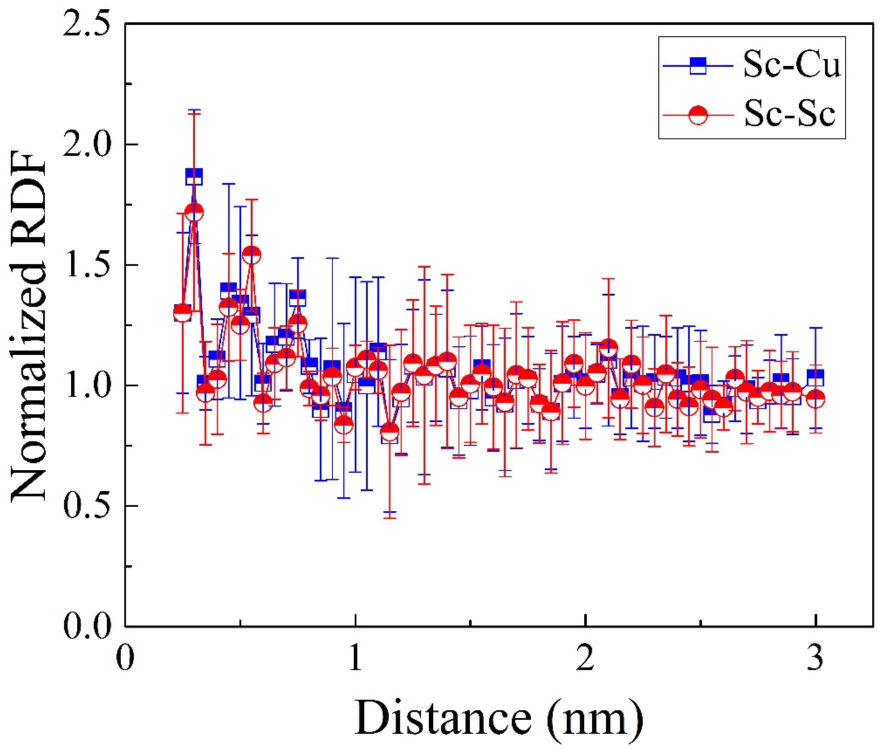

Supplementary Figure 9 | The partial radial distribution function (RDF) curves of Cu and Sc atoms around Sc atoms in the AlCuSc-C alloy after natural aging for 6 months. Around Sc atoms, the concentrations of both Cu and Sc atoms are higher within a radial distance of ~ 1.0 nm than longer distances. The error bars are standard deviations of the mean.

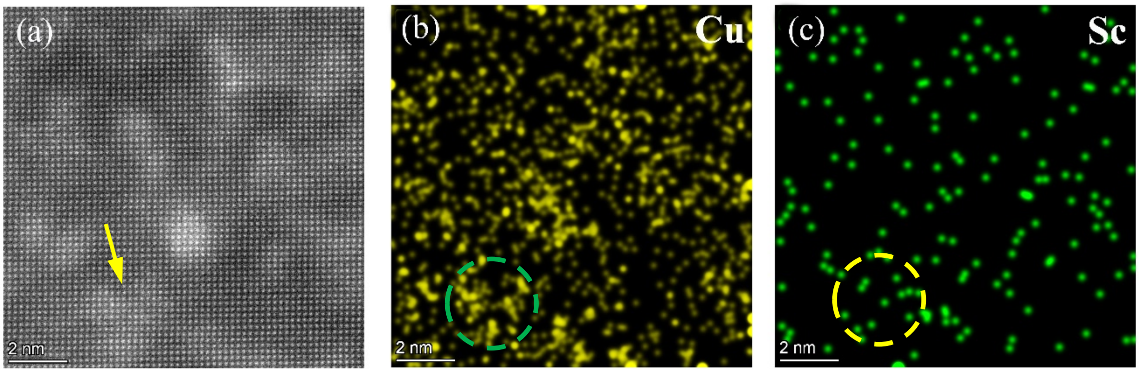

Supplementary Figure 10 | Cu and Sc rich solute complexes in the naturally aged AlCuSc-C alloy. Typical HAADF-STEM image viewed along <100>$_{Al}$ showing the solute complexes (**a)** and the corresponding EDS mapping of Cu (**b)** and Sc **(c).** The region marked by the arow in **a** is a solute complex, which is rich in Cu and slightly rich in Sc.

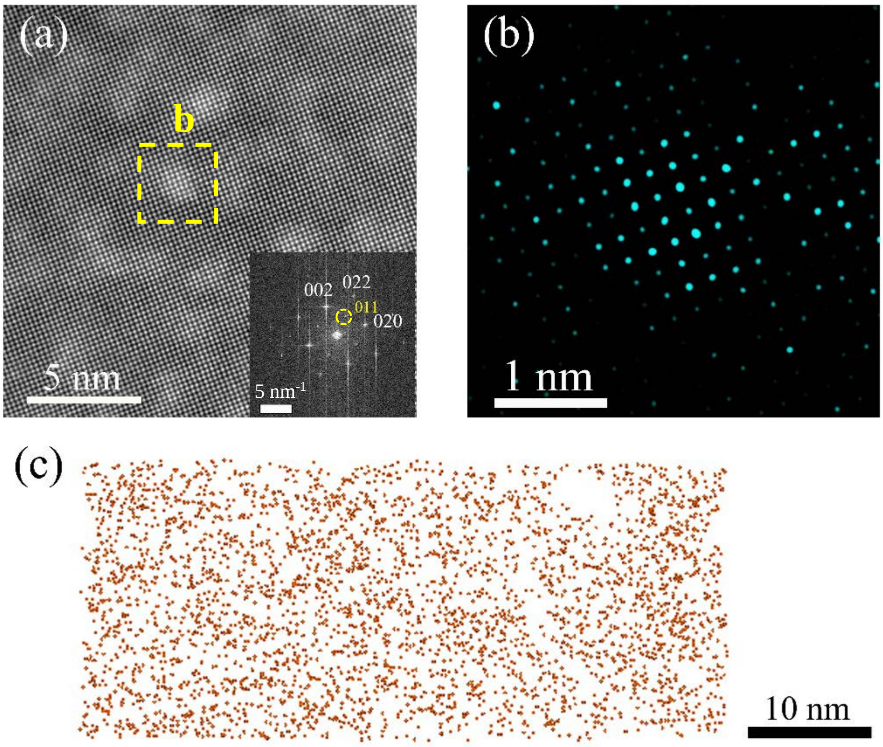

Supplementary Figure 11 | Solute complexes in the naturally aged AlCuSc-C alloy. a, Typical HAADF-STEM image viewed along <100>$_{Al}$ showing the solute complexes, the region marked by the dashed square shows a typical solute complex. **b,** Inverse FFT image of the framed atom complex in **a**, after Gaussian filtering, where no clear periodic arrangement of the high contrast atom columns enriched with Cu and Sc can be observed. **c,** Thin slice from the three-dimensional reconstruction of a matrix volume with a thickness of 6 nm in an APT dataset showing the distribution of Cu atoms, which confirms that no apparently large clusters exist.

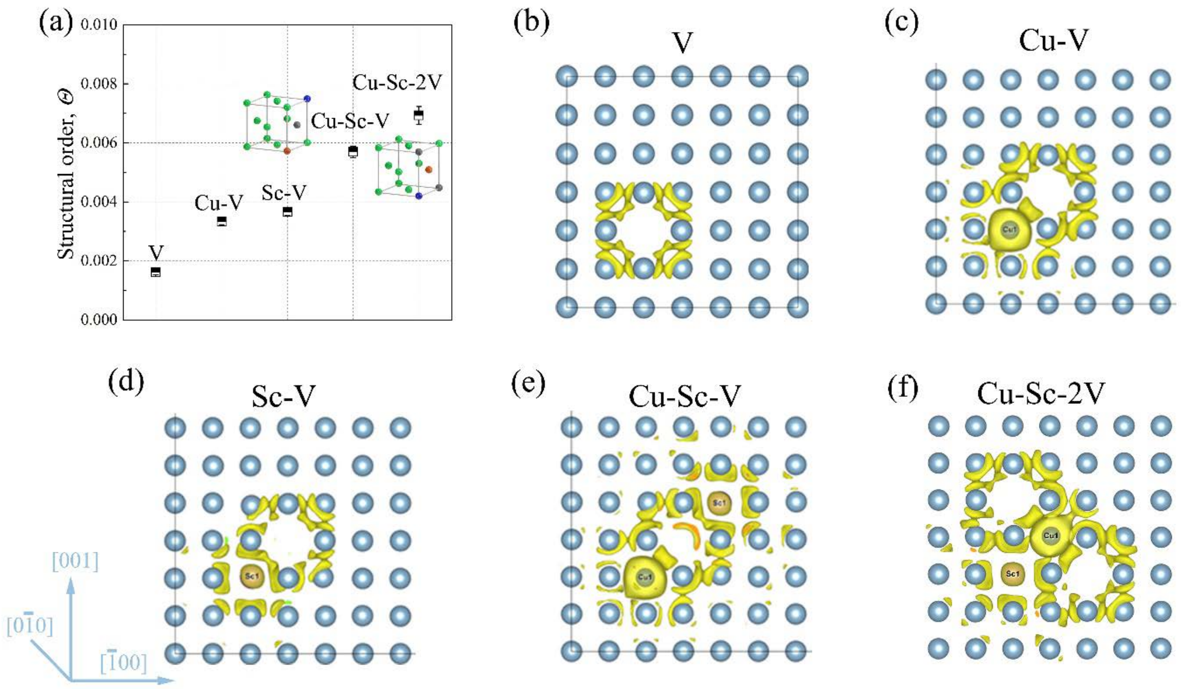

Supplementary Figure 12 | The physical origin of the enhanced binding energy between Cu and Sc atoms in a complex. a, Structural order parameter Θ of V, Cu-V, Sc-V, Cu-Sc-V and Cu-Sc-2V. Isoconcentration surface of charge density 0.0335 e·Bohr^{-3} in V **(b)**, Cu-V **(c)**, Sc-V **(d)**, Cu-Sc-V **(e)** and Cu-Sc-2V **(f)**. The definition of structural order parameter Θ obeys that in ref[1]. Adding solute atom and vacancy increase Θ, indicative of larger lattice distortion. With vacancy being involved, the charge density significantly increases. Charge accumulation and depletion would result in strengthening and weakening of interatomic bonds, respectively[2]. Thus, the enhanced binding energy in V-Sc-Cu-V atom complex originates from the increased charge density.

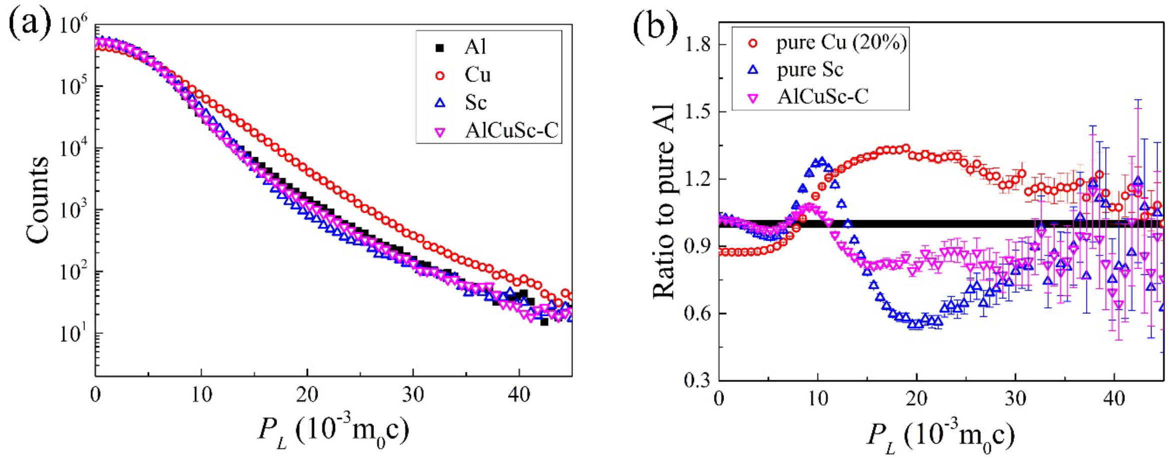

Supplementary Figure 13 | CDB spectra and ratio curves for pure Al, Cu, Sc and the AlCuSc-C alloy. a, spectra**. b,** ratio curves for pure Cu reduced to 20% in amplitude, pure Sc, and the AlCuSc-C alloy normalized to pure Al. The error bars are standard deviations of the mean. P_L is the longitudinal component of the positron-electron momentum along the direction of the γ-ray emission, c is the speed of light, m_0 is the electron rest mass.

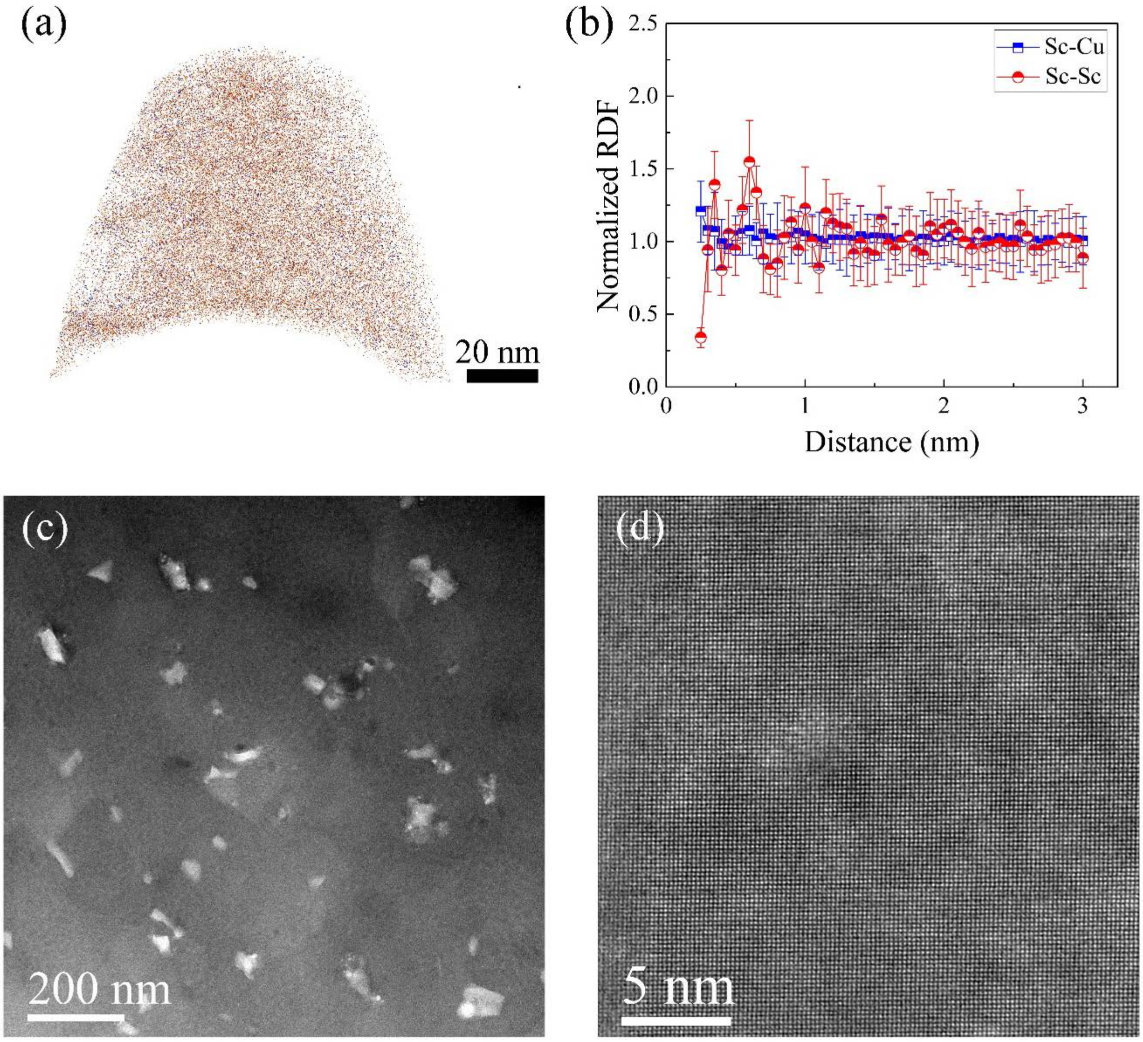

Supplementary Figure 14 | APT analysis of the as-processed AlCuSc-R alloy and HAADF-STEM images of the AlCuSc-R alloy stored at room temperature for 6 months. a, Representative APT reconstruction of the as-processed AlCuSc-R alloy, where Cu and Sc atoms are labelled with brown and blue colors, respectively. **b,** The partial radial distribution function (RDF) curves of Cu and Sc atoms around Sc atoms in the as-processed AlCuSc-R alloy. The error bars are standard deviations of the mean. **c,** Typical HAADF-STEM image of the AlCuSc-R alloy showing an apparent θ precipitation. **d,** High-resolution HAADF-STEM image viewed along $<100>_{Al}$ showing no apparent (Cu, Sc, vacancy)-rich atomic complexes in the AlCuSc-R alloy. The RDF analysis in **b** shows a slight enrichment of Cu atoms around Sc atoms, implying that (Cu, Sc, vacancy)-rich atomic complexes have also formed during room-temperature HPT. A comparison to the RDF map in Supplementary Fig. 9 shows that the normalized concentration of Cu around Sc atoms is much lower than that of the AlCuSc-C sample, indicating that the number density of (Cu, Sc, vacancy)-rich atomic complexes in the AlCuSc-R alloy is much lower.

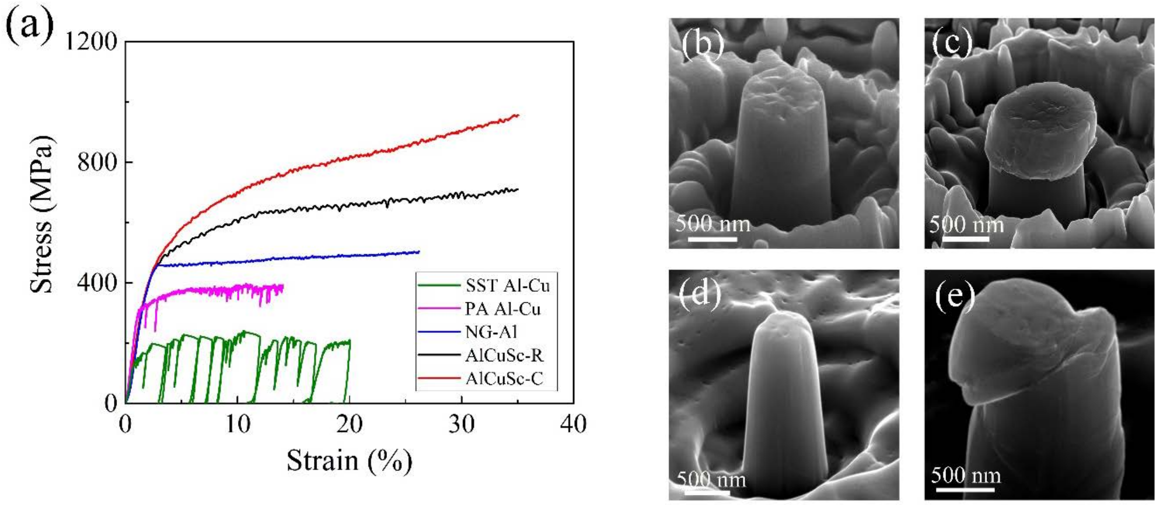

Supplementary Figure 15 | Compressive curves and deformation morphology of micropillars. a, Compressive engineering stress-strain curves of the solid solution heat treated (SST) Al-Cu micropillar, peak-aged (PA) coarse-grained Al-Cu micropillar with plate-like θ' precipitates, NG-Al (fabricated by magnetron sputtering) micropillar, and the AlCuSc-R and AlCuSc-C micropillars. **b** and **c,** Fabricated 1μm-diameter NG-Al micropillar and the deformation morphology after compression. **d and e,** As-fabricated 1μm-diameter solid solution heat treated Al-Cu micropillar and the morphology after compression.

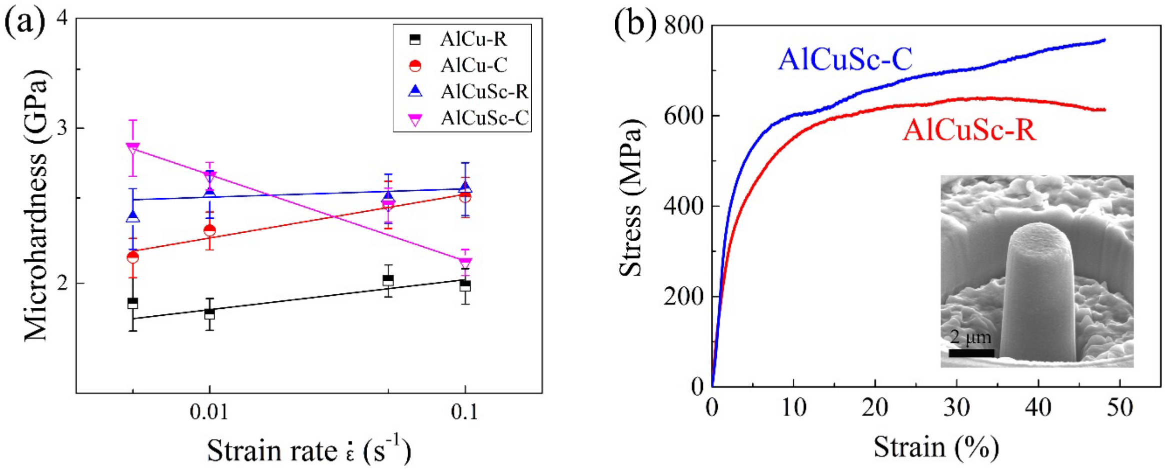

Supplementary Figure 16 | Mechanical properties obtained from nanoindentation and micropillar compression. a, The measured microhardness as a function of strain rate for the AlCu-R, AlCu-C, AlCuSc-R and AlCuSc-C alloys. The error bars represent standard deviations from the mean for sets of six tests. b**,** Compressive engineering stress-strain curves of 3 μm-diameter AlCuSc-R and AlCuSc-C micropillars at a strain rate of 2×10^{-4} s^{-1}.

Supplementary Table 1 | Positron annihilation lifetimes, fraction of vacancy-type defects, number density of vacancies and vacancy concentration of the AlCu-R, AlCu-C, AlCuSc-R and AlCuSc-C alloys.

Alloys	Positron annihilation lifetimes, τ(ps)	Fractions of vacancy-type defects			Number density of vacancies (10^{23} m^{-3})	Vacancy concentration (at.%)
		Vacancies associated with dislocations ($f1$) %	Monovacancies in the bulk ($f2$) %	Divacancies in the bulk ($f3$) %		
AlCu-R	228	68	32		19.2±1.5	0.0032
AlCu-C	226	76	24		20.5±2	0.0036
AlCuSc-R	236	36	62		62±5	0.01
AlCuSc-C	258		53.5	46.5	1300±100	0.22

Note: In the AlCu-R, AlCu-C and AlCuSc-R alloys, the fractions of the vacancy-type defects can be estimated by τ=220f_1+245f_2^{3}, where f_1 is the fraction of vacancies associated with dislocations and f_2 is the fraction of bulk monovacancies. In the AlCuSc-C alloy, the fractions of the vacancy-type defects can be estimated by τ=245f_2+273f_3, where f_2 is the fraction of bulk monovacancies and f_3 is the fraction of bulk divacancies.

Supplementary References:

1 Tong, H. & Tanaka, H. Role of Attractive Interactions in Structure Ordering and Dynamics of Glass-Forming Liquids. *Phys. Rev. Lett.* **124**, 225501 (2020).

2 Zhao, D., Løvvik, O., Marthinsen, K. & Li, Y. Segregation of Mg, Cu and their effects on the strength of Al S5 (210) [001] symmetrical tilt grain boundary. *Acta Mater.* **145**, 235-246 (2018).

3 Su, L. H., Lu, C., He, L. Z., Zhang, L. C. & Li, H. J. Study of vacancy-type defects by positron annihilation in ultrafine-grained aluminum severely deformed at room and cryogenic temperatures. *Acta Mater.* **60**, 4218-4228 (2012).
